# Glutamyl-Prolyl-tRNA Synthetase Regulates Epithelial Expression of Mesenchymal Markers and Extracellular Matrix Proteins: Implications for Idiopathic Pulmonary Fibrosis

**DOI:** 10.3389/fphar.2018.01337

**Published:** 2018-11-20

**Authors:** Dae-Geun Song, Doyeun Kim, Jae Woo Jung, Seo Hee Nam, Ji Eon Kim, Hye-Jin Kim, Jong Hyun Kim, Cheol-Ho Pan, Sunghoon Kim, Jung Weon Lee

**Affiliations:** ^1^Department of Pharmacy, Research Institute of Pharmaceutical Sciences, College of Pharmacy, Seoul National University, Seoul, South Korea; ^2^Systems Biotechnology Research Center, Korea Institute of Science and Technology (KIST), Gangneung-si, South Korea; ^3^Medicinal Bioconvergence Research Center, Seoul National University, Seoul, South Korea; ^4^Interdisciplinary Program in Genetic Engineering, Seoul National University, Seoul, South Korea

**Keywords:** idiopathic pulmonary fibrosis, bleomycin fibrotic animal model, extracellular matrix, prolyl-tRNA-synthetase, signal transduction, STAT6

## Abstract

Idiopathic pulmonary fibrosis (IPF), a chronic disease of unknown cause, is characterized by abnormal accumulation of extracellular matrix (ECM) in fibrotic foci in the lung. Previous studies have shown that the transforming growth factor β1 (TGFβ1) and signal transducers and activators of transcription (STAT) pathways play roles in IPF pathogenesis. Glutamyl-prolyl-tRNA-synthetase (EPRS) has been identified as a target for anti-fibrosis therapy, but the link between EPRS and TGFβ1-mediated IPF pathogenesis remains unknown. Here, we studied the role of EPRS in the development of fibrotic phenotypes in A549 alveolar epithelial cells and bleomycin-treated animal models. We found that EPRS knockdown inhibited the TGFβ1-mediated upregulation of fibronectin and collagen I and the mesenchymal proteins α-smooth muscle actin (α-SMA) and snail 1. TGFβ1-mediated transcription of collagen I-α1 and laminin γ2 in A549 cells was also down-regulated by EPRS suppression, indicating that EPRS is required for ECM protein transcriptions. Activation of STAT signaling in TGFβ1-induced ECM expression was dependent on EPRS. TGFβ1 treatment resulted in EPRS-dependent *in vitro* formation of a multi-protein complex consisting of the TGFβ1 receptor, EPRS, Janus tyrosine kinases (JAKs), and STATs. *In vivo* lung tissue from bleomycin-treated mice showed EPRS-dependent STAT6 phosphorylation and ECM production. Our results suggest that epithelial EPRS regulates the expression of mesenchymal markers and ECM proteins via the TGFβ1/STAT signaling pathway. Therefore, epithelial EPRS can be used as a potential target to develop anti-IPF treatments.

## Introduction

Idiopathic pulmonary fibrosis (IPF) is a chronic, progressive, fatal, fibrotic interstitial lung disease of unknown cause (Zhou et al., 2017; Bai et al., 2018; Lederer and Martinez, 2018; Milara et al., 2018). Typical clinical symptoms include dyspnoea, decreased exercise capacity, and dry cough; most patients survive for 2.5–5 years after diagnosis (Raghu et al., 2015). Idiopathic pulmonary fibrosis (IPF) is characterized by the excessive accumulation of extracellular matrix (ECM) components, which correlates with the proliferation and activation of fibroblasts, myofibroblasts, and abnormal lung epithelial cells (Wolters et al., 2014). Although the origins and activation of invasive lung myofibroblasts remain unclear, some potential causes include activation of lung resident fibroblasts, recruitment of circulating fibrocytes, and blood mesenchymal precursors; and mesenchymal transformation of alveolar type II epithelial cells, endothelial cells, pericytes, and/or mesothelial cells (Bagnato and Harari, 2015).

Current pharmacologic treatments for IPF include two U.S. Food & Drug Administration-approved drugs (nintedanib and pirfenidone) that improve symptoms but do not cure the disease (Lederer and Martinez, 2018). Given the limited treatment options, it is urgent to investigate the mechanisms of IPF pathogenesis (Milara et al., 2018).

Transforming growth factor β1 (TGFβ1) is a multifunctional cytokine that regulates immune responses during homeostasis and inflammation (Luzina et al., 2015). During IPF pathogenesis, TGFβ1 activates lung fibroblasts and promotes epithelial mesenchymal transformations (EMT) of various cell types, such as alveolar type II cells (Ghosh et al., 2013; Milara et al., 2018). Disrupting TGFβ1-mediated signaling will be important to develop effective anti-fibrogenesis drugs.

Prolyl-tRNA synthetase (PRS) catalyzes the attachment of proline to transfer RNA (tRNA) during translation. Halofuginone (HF), a plant alkaloid isolated from *Dichroa febrifuga* (Keller et al., 2012), is an anti-fibrotic agent that blocks PRS catalytic activity. HF inhibits mRNA levels of collagens, *COL1A1* (with 19% proline/total residues) and *COL1A2*, but this effect is reversed by exogenous proline (Keller et al., 2012). HF also blocks non-translational functions of PRS, such as inhibiting synthesis of fibronectin 1 (with 7.9% proline/total residues), an ECM protein that is not proline-rich. HF-mediated inhibition of PRS leads to the accumulation of naked tRNA molecules, which activates the amino-acid response (AAR) pathway to inhibit the synthesis of ECM proteins. Such HF-mediated inhibition of PRS and ECM expression are overcame by exogenous proline treatment, indicating that PRS can be involved in ECM translation via proline charging of prolyl-tRNA (Keller et al., 2012). However, it may still be likely that roles of PRS in ECM expression involve non-translational processes, since variable ECMs can be composed with different levels of proline.

Studies have demonstrated a role for the Janus kinase (JAK)-signal transducer and activator of transcription (STAT) pathway in IPF. STAT3 is activated in the lungs of patients with IPF (Pechkovsky et al., 2012; Prele et al., 2012; Pedroza et al., 2016). TGFβ receptor 1 (TGFβR1) forms a protein complex with JAK1 that activates STAT3 via SMAD3 meditation (Tang et al., 2017). STAT3 is essential for activation of the COL1A2 enhancer (Papaioannou et al., 2018). A link between STAT3/STAT6 and IPF has also been reported (Nikota et al., 2017; Milara et al., 2018). However, the role of EPRS in TGFβ1/STAT signaling-induced IPF pathogenesis remains.

Here, we studied the functional role of EPRS in TGFβ1-mediated fibrosis. We found that EPRS activated TGFβ1-induced ECM protein expression both *in vitro* and *in vivo*. TGFβ1 treatment resulted in the formation of a multi-protein complex consisting of TGFβR1, EPRS, JAKs, and STATs in alveolar type II epithelial cells. EPRS-dependent STAT6 phosphorylation correlated with ECM production in the lungs of bleomycin-treated mice. Our results suggested that epithelial EPRS regulates TGFβ/STAT signaling to induce expression of mesenchymal markers and ECM proteins during IPF development.

## Materials and methods

### Reagents and plasmids

All cytokines and growth factors including TGFβ1 were purchased from Peprotech (Rocky Hill, NJ, United States). Hydroxyproline assay kits, and CCl_4_ were purchased from Sigma-Aldrich (St. Louis, MO, United States). Bleomycin and target specific pooled siRNAs siSTAT3 and siSTAT6 were purchased from Santa Cruz Biotechnology (Santa Cruz, CA, United States). EPRS in pEXPR-103-Strep vector (IBA Lifesciences, Göettingen, Germany) were gifts from Dr. Myung Hee Kim at the Korea Research Institute of Bioscience and Biotechnology (KRIBB, Daejeon, Korea). EPRS (1–1440 amino acids) consists of ERS (1–687 amino acids) and PRS (935–1,440 amino acids) linked via non-catalytic WHEP repeat domains (688–934 amino acids) (Ray and Fox, 2014). The PRS domain of EPRS was cloned into pEXPR-103-Strep vector (IBA Lifesciences). pRc/CMV-WT STAT3 was previously described (Choi et al., 2009) and pCMV-STAT6-IRES-Neo was a gift from Axel Nohturfft (Addgene plasmid # 35482). Adenovirus expressing SMAD2 or SMAD3 were described previously (Lee et al., 2005).

### Cell culture

A549 lung adenocarcinoma cells were purchased from the Korean Cell Line Bank (KCLB, Seoul, Korea) and cultured in RPMI (SH30027.01, Hyclone, South Logan, UT, United States). Media were supplemented with 10% fetal bovine serum (FBS, GenDEPOT, Barker, TX, United States) and 1% penicillin/streptomycin (GenDEPOT) and cells were grown at 37°C in 5% CO_2_. The SMARTvector shEPRS doxycycline-inducible knockdown cell line was established by treating lentiviral particles (EPRS mCMV-turboGFP V2IHSMCG_687815, 687823, Dharmacon, Lafayette, CO, United States). Positive clones were enriched by treatment of 2 μg/ml puromycin (GenDEPOT) and maintained in complete media supplemented with 1 μg/ml puromycin. siRNAs or cDNA plasmids were transiently transfected using Lipofectamine RNAiMAX or Lipofectamine 3000, respectively, following the manufacturer's instructions (Thermo Fisher Scientific, Waltham, MA, United States).

### Western blot analysis

Subconfluent cells or animal tissues were harvested for whole cell or tissue extracts using RIPA buffer. Proteins in the lysates were separated in Tris-Glycine SDS-polyacrylamide gels at concentrations ranging from 8 to 12%, and transferred to nitrocellulose membranes (Thermo Fisher Scientific). Target-specific antibodies used in this study are summarized in Table 1 (Supplementary Data Sheet 1).

**Table 1:** Antibodies and their dilution ratio used in this study.

**Name**	**Company**	**Catalog**	**WB dil**.	**IHC dil**.
EPRS	Neomics	NMS-01-0004	1:5,000	1:200
Fibronectin	DAKO	A0245	1:5,000	1:200
Collagen I	Acris	R1038X	1:1,000	1:200
β-actin	Abcam	AB133626	1:1,000	–
pY^641^STAT6	Abcam	AB28829	1:1,000	1:100
STAT6	Cell signaling technology	#9362	1:1,000	–
pY^705^STAT3	Abcam	AB76315	1:1,000	–
pY^705^STAT3	Cell signaling technology	#9145	–	1:200
STAT3	Santa cruz biotechnology	SC-482	1:1,000	–
pS^465/467^SMAD2	Cell signaling technology	#3108	1:1,000	1:100
SMAD2	Cell signaling technology	#5339	1:1,000	–
pS^423/425^SMAD3	Cell signaling technology	#9520	1:1,000	–
SMAD3	Cell signaling technology	#9523	1:1,000	–
TGFβ1-Receptor	Santa cruz biotechnology	SC-399	1:500	–
JAK1	Cell signaling technology	#3344	1:1,000	–
JAK2	Millipore	04-001	1:1,000	–
STAT1	Santa cruz biotechnology	SC-346	1:1,000	–
STAT5	Santa cruz biotechnology	SC-835	1:1,000	–
KRS	Neomics	NMS-01-0005	1:2,000	–
ERKs	Cell signaling technology	#9102	1:1,000	–
Anti-Strep	IBA life sciences	2-1509-001	1:2,500	–
α-SMA	Sigma	A2547	–	1:200
Snail 1	Cell signaling technology	#3895	1:1,000	–
Laminin γ2	Santa cruz biotechnology	SC-28330	–	1:200
Laminins	Abcam	AB11575	1:1,000	–

### qRT-PCR

Total RNAs from animal tissues or cells were isolated using Qiazol Reagent (Qiagen, Hilden, Germany), and their cDNAs were synthesized using amfiRivert Platinum cDNA synthesis master mix (GenDEPOT) according to the manufacturer's instructions. Quantitative real time PCR (q-PCR) samples were prepared with LaboPassTM EvaGreen Q Master (Cosmo Genetech, Seoul, Korea) prior to analysis in a CFX Connect™ Real-Time PCR machine (Bio-Rad, Hercules, CA, United States). mRNA levels were normalized against GAPDH and CFX Maestro™ software (Sunnyvale, CA, United States) was used to analyse the data. Primers were purchased from Cosmo Genetech (Seoul, Korea). The primer sequences are shown in Table 2.

**Table 2:** qRT-PCR primers used in this study.

**Gene name**	**Forward**	**Reverse**	**Size (bp)**
Human EPRS	AGGAAAGACCAACACCTTCTC	CTCCTTGAACAGCCACTCTATT	87
Human collagen1A1	CAGACTGGCAACCTCAAGAA	CAGTGACGCTGTAGGTGAAG	97
Human DDIT3	GAGATGGCAGCTGAGTCATT	TTTCCAGGAGGTGAAACATAGG	134
Human collagen4A1	CGGGCCCTAAAGGAGATAAAG	GAACCTGGAAACCCAGGAAT	115
Human fibronectin	CCACAGTGGAGTATGTGGTTAG	CAGTCCTTTAGGGCGATCAAT	104
Human laminin γ2	CTCAGGAGGCCACAAGATTAG	TGAGAGGGCTTGTTTGGAATAG	101
Mouse collagen 1A1	AGACCTGTGTGTTCCCTACT	GAATCCATCGGTCATGCTCTC	113
Mouse fibronectin	TCCTGTCTACCTCACAGACTAC	GTCTACTCCACCGAACAACAA	96
Mouse laminin γ2	TGGAGTTTGACACGGATAAGG	GAGTGTGTCTTGGATGGTAACT	104

### Co-immunoprecipitation

Whole-cell lysates were prepared using immunoprecipitation lysis buffer (40 mM HEPES pH7.4, 150 mM NaCl, 1 mM EDTA, 0.5% Triton X-100, and protease inhibitors) and precipitated with Pierce High-Capacity Streptavidin Agarose beads (Thermo Fisher Scientific) overnight at 4°C. Precipitates were washed three times with ice-cold lysis buffer, three times with immunoprecipitation wash buffer (40 mM HEPES pH 7.4, 500 mM NaCl, 1 mM EDTA, 0.5% Triton X-100, and protease inhibitors), and then boiled in 2 × SDS-PAGE sample buffer before immunoblotting.

### Luciferase assay

To analyze promoter activity, *LAMC2* (laminin γ2) promoters (encoding regions of −1871 to +388) and *COL1A1* (collagen I α1) promoters (encoding regions of −2865 to +89) were amplified by PCR and cloned into the pGL3-basic vector. A549 cells were seeded in 48 well plates and the next day the plasmids were transfected using Lipofectamine 3000 transfection reagent (Thermo Fisher Scientific). β-Gal was co-transfected to allow normalization. One day after transfection, TGFβ1 (2 ng/ml) was added to the culture media. After 24 h, luciferase activity was measured according to the manufacturer's instructions using a luciferase reporter assay kit (Promega, Madison, WI, United States) with a luminometer (DE/Centro LB960, Berthold Technologies, Oak Ridge, TN, United States).

### Animal experiments

Wildtype (WT) *EPRS*^+/+^ (*n* = 4 for vehicle and *n* = 9 for bleomycin) and *EPRS*^−/+^ hetero-knockout (*n* = 5 for vehicle and *n* = 7 for bleomycin) C57BL/6 mice were housed in a specific pathogen-free room with controlled temperature and humidity. Mouse protocol and animal experiments were approved by the Institutional Animal Care and Use Committee (IACUC) of Seoul National University (SNU-161201-1-3). For the lung fibrosis model, bleomycin (Santa Cruz Biotechnology) was dissolved in sterilized saline and intratracheal instillation was performed through surgically exposed trachea as a single dose of 1 mg/kg in 100 μl solution per animal. Mice were sacrificed 4 weeks post-intratracheal instillation. Lung tissue samples were snap frozen in liquid nitrogen for western blot, qPCR, and hydroxyproline analysis, or fixed in 4% formaldehyde in PBS for histological analysis.

### Immunohistochemistry and staining

Paraffin blocks and sections (6-μm thickness) of lung tissues were prepared by Abion Inc. (Seoul, Korea) for immunohistochemistry analysis. Primary antibodies and their dilution ratios are listed in Table 1. Vectastain ABC-HRP kit (Vector Laboratories, Burlingame, CA, United States) were used to visualize the stained samples. Mayer's hematoxylin (Sigma-Aldrich) was used for counter-staining the nuclei.

### Statistics

Statistical analyses were performed using Prism software version 6.0 (GraphPad, La Jolla, CA, United States). Two-way analysis of variance (ANOVA) in group analysis or Student's *t*-tests were performed to determine statistical significance. A value of *p* < 0.05 was considered significant.

## Results

### EPRS expression regulated ECM production in A549 alveolar type II cells upon TGFβ1 stimulation

**Figure 1:**
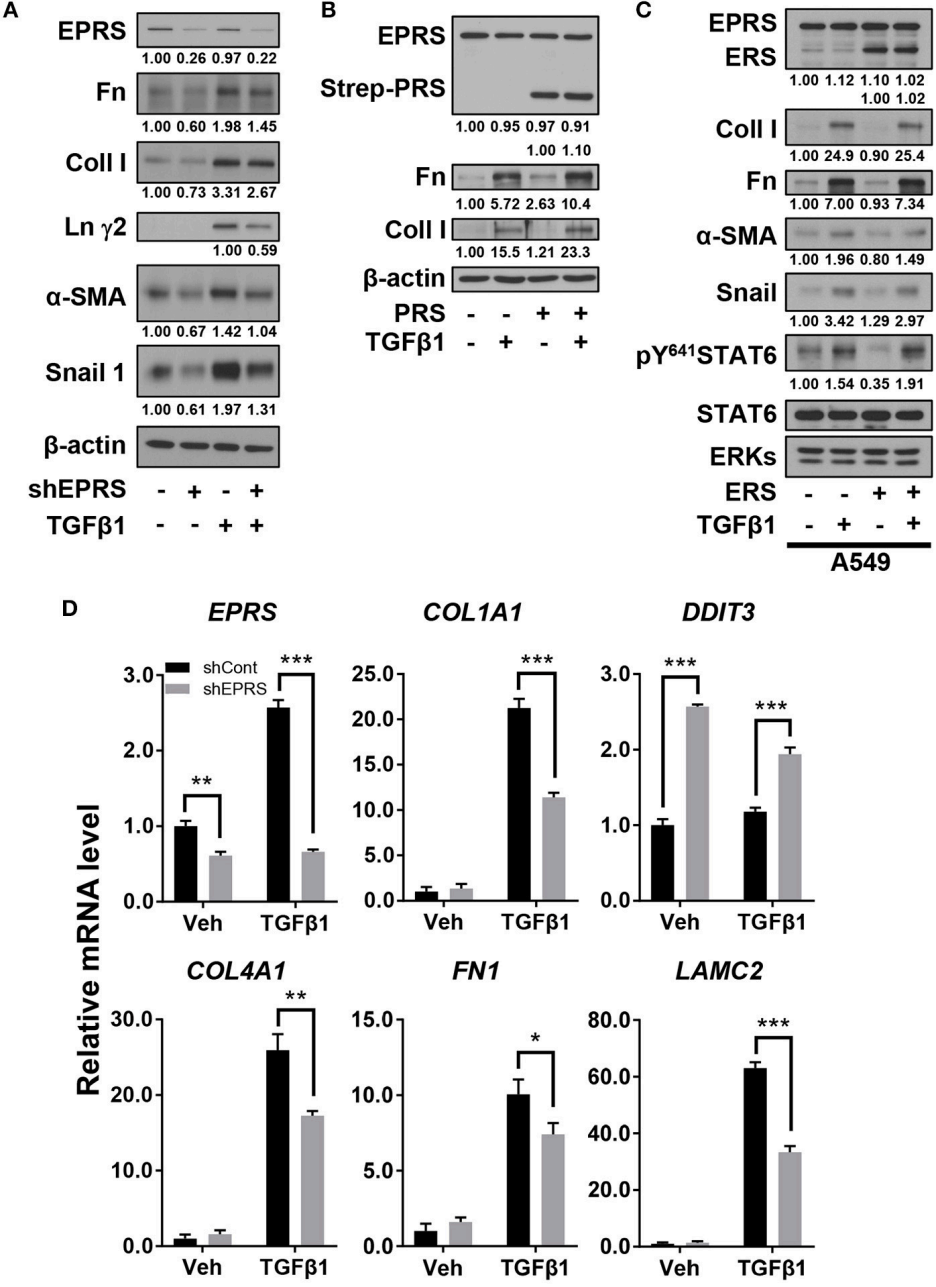
EPRS expression regulated ECM protein production in A549 alveolar type II cells treated with TGFβ1. **(A,B)** A549-control (–) or shEPRS doxycycline-inducible knockdown (+) A549 cell line or control A549 cells transiently transfected with PRS expression vector (pEXPR-103-Strep-PRS) were treated without or with TGFβ1 (2 ng/ml) for 24 h, and harvested for immunoblottings for the indicated molecules. **(C)** A549 cells were transfected without or with ERS expression plasmid for 24 h and then treated without or with TGFβ1 (2 ng/ml) for 24 h before lysate preparation and immunoblotting. **(D)** Subconfluent control (shCont) or shEPRS-A549 cells were treated with TGFβ1 (2 ng/ml) for 24 h, before qRT-PCR analysis. Data are presented at mean ± standard deviation (SD). ^*^, ^**^, and ^***^ indicate significance at *p* < 0.05, 0.01, and 0.001, respectively (calculated by Student's *t*-tests). Data shown represent three independent experiments.

We studied the regulatory effect of EPRS on the expression of different ECM proteins by introducing doxycycline-inducible EPRS knockdown vectors into the A549 alveolar type II cell line. Expression of ECM proteins such as collagen I, fibronectin, and laminin γ2 were tested by immunoblotting EPRS-knockdown and control A549 cells. All the ECM proteins showed increased expression in control cells treated with TGFβ1 and this effect was abolished in EPRS-knockdown cells (Figure 1A). Expression levels of mesenchymal proteins including α-smooth muscle actin (α-SMA) and snail 1 were also dependent on TGFβ1 treatment and/or EPRS expression (Figure 1A). Since EPRS protein consists of two glutamyl-tRNA-synthetase (ERS) and prolyl-tRNA-synthetase (PRS), it would be reasonable to see whether PRS alone could achieve these effects. Overexpression of PRS enhanced TGFβ1-induced ECM protein expression (Figure 1B). However, overexpression of ERS alone did not increase TGFβ1-mediated ECM protein expression (Figure 1C), indicating that the PRS component of EPRS regulates ECM protein expression. TGFβ1 treatment also increased mRNA levels of *COL1A1, COL4A1, FN1*, and *LAMC2* in control cells, while EPRS suppression inhibited this effect (Figure 1D). Our results suggest that EPRS positively regulated TGFβ1-induced expression of ECM proteins. A previous report (Keller et al., 2012) stated that EPRS suppression increases mRNA levels of DNA damage-inducible transcript 3 (*DDIT3*, also known as *CHOP*) to indicate an activation of AAR pathway that supports for tRNA charging processes. However, we found that *DDIT3* mRNA levels were unaffected by TGFβ1 treatment, indicating that TGFβ1-induced regulation of ECM protein expression involves alternative mechanism(s) in addition to the role in tRNA charging (Figure 1D).

### Regulation of TGFβ1-induced ECM protein synthesis by EPRS occurred via STAT activation

**Figure 2:**
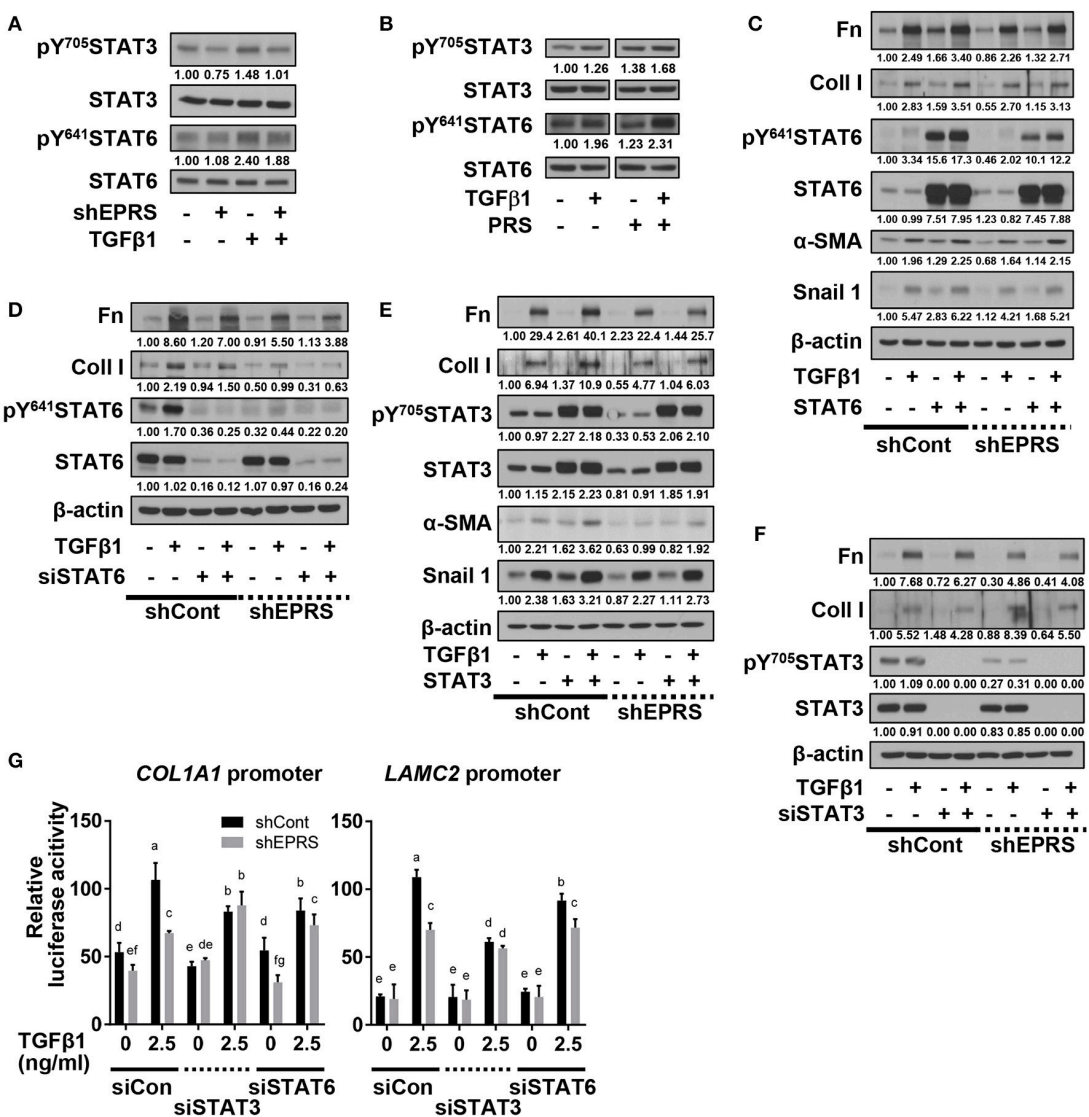
Regulation of TGFβ1-induced ECM protein synthesis by EPRS occurred via STAT activation. **(A–G)** A549 cells stably infected with control (–) or shEPRS virus (A549-shEPRS) or control A549 cells transiently transfected with different expression vectors or siRNAs as indicated were treated without (–) or with TGFβ1 (2 ng/ml, +) for 24 h, before whole cell extracts preparation and immunoblotting for the indicated molecules. **(G)** A549 cells transfected with *COL1A1* or *LAMC2* promoter-luciferase constructs with STATs-consensus responsive sequences (*Col1a1*-2.9 and *Lamc2*-2.3 kb constructs with upstream promoter regions up to −2.9 and −2.3 kb, respectively) together with either siRNA against control sequence (siCon), STAT3 (siSTAT3), or STAT6 (siSTAT6) were treated with TGFβ1 (0 or 2.5 ng/ml) for 24 h, prior to luciferase reporter analysis. Data are presented as mean ± SD. Different letters indicate statistical significance at *p* < 0.05 according to one-way ANOVA. Data shown are from three isolated experiments.

To investigate potential signaling molecules or pathways involved in EPRS-mediated regulation of ECM protein synthesis following TGFβ1-treatment, we studied the dependency of STAT3 and STAT6, known mediators of IPF (Nikota et al., 2017; Milara et al., 2018), on EPRS expression. We found that TGFβ1 promoted the phosphorylation of STAT3 at Tyr705 (pY^705^STAT3) and STAT6 at Tyr641 (pY^641^STAT6), and these effects were abolished by EPRS suppression (Figure 2A). EPRS overexpression increased pY^705^STAT3 and pY^641^STAT6 levels upon TGFβ1 treatment in A549 cells (Figure 2B). Our results suggest that EPRS and TGFβ1 signaling regulate STAT3 and STAT6 phosphorylation. We studied the role of EPRS in STAT-mediated expression of ECM proteins. Overexpression of STAT6 indicate greater increases in basal and TGFβ1-induced levels of α-SMA, snail 1, fibronectin, and collagen I in EPRS-positive A549 cells compared with EPRS-knockdown cells (Figure 2C). However, basal and TGFβ1-induced expression levels of fibronectin and collagen I were decreased when STAT6 levels were suppressed (Figure 2D). A similar EPRS-dependent regulation pattern of fibronectin and collagen I was observed when STAT3 was modulated (Figures 2E,F). We also tested the transcriptional activities of *COL1A1* and *LAMC2* promoters in A549 cells lacking STAT3 or STAT6. *COL1A1* or *LAMC2* promoters containing STAT-responsive consensus sequences showed increased transcriptional activity in A549 cells treated with TGFβ1. However, EPRS suppression reduced these effects (Figure 2G). Suppression of STAT3 or STAT6 abolished the increased transcriptional activity of *COL1A1* or *LAMC2* in EPRS-positive A549 cells but not EPRS-suppressed cells (Figure 2G). Together, our results suggested that EPRS regulates ECM protein expression via STAT3 or STAT6 signaling induced by TGFβ1.

### TGFβ1-mediated SMAD3 phosphorylation upregulated phosphorylation of STAT6 depending on EPRS expression

**Figure 3:**
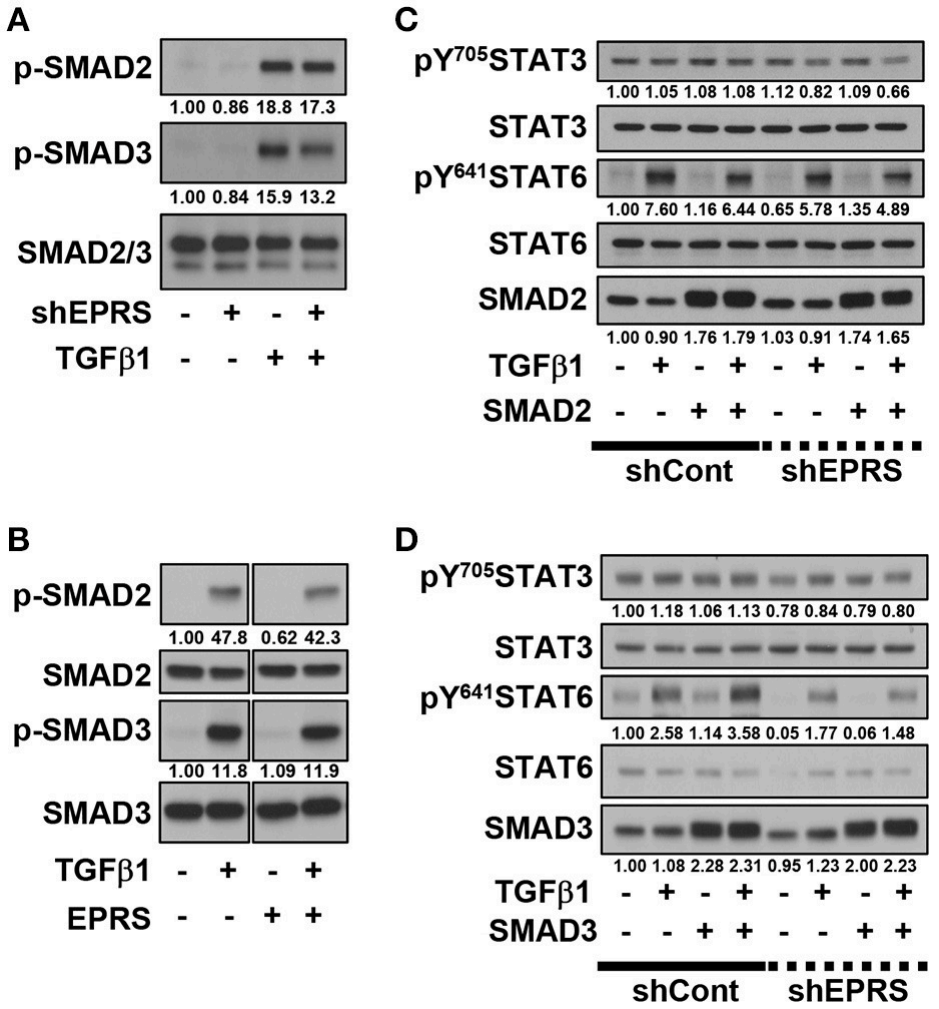
TGFβ1-mediated SMAD3 phosphorylation upregulated phosphorylation of STAT6 depending on EPRS expression. **(A–D)** A549-shControl (–) or A549-shEPRS (+) cells were treated with vehicle (–) or TGFβ1 (2 ng/ml, +) **(A,B)** or treated with vehicle (–) or TGFβ1 (2 ng/ml, +) after infection (24 h) with adenovirus encoding for SMAD2 or SMAD3 (**C**,**D** respectively). The whole cell extracts were then prepared before normalization and immunoblotting for the indicated molecules. Data shown represent three independent experiments.

We investigated the role of the TGFβ1-mediated SMAD signaling in EPRS-dependent ECM protein expression and STAT3/6 activity. TGFβ1-mediated SMAD2 and SMAD3 phosphorylation was partially inhibited by EPRS suppression (Figure 3A). However, EPRS overexpression did not affect the levels of phosphorylated SMAD2 or SMAD3, which might have already been saturated by TGFβ1 treatment (Figure 3B). TGFβ1-induced levels of pY^641^STAT6 were increased by overexpression of SMAD3 but not SMAD2. EPRS suppression abolished that effect (Figures 3C,D).

### EPRS-mediated signaling in TGFβ1-treated cells involved the formation of a multi-protein complex consisting of STAT6 and TGFβ1R

**Figure 4:**
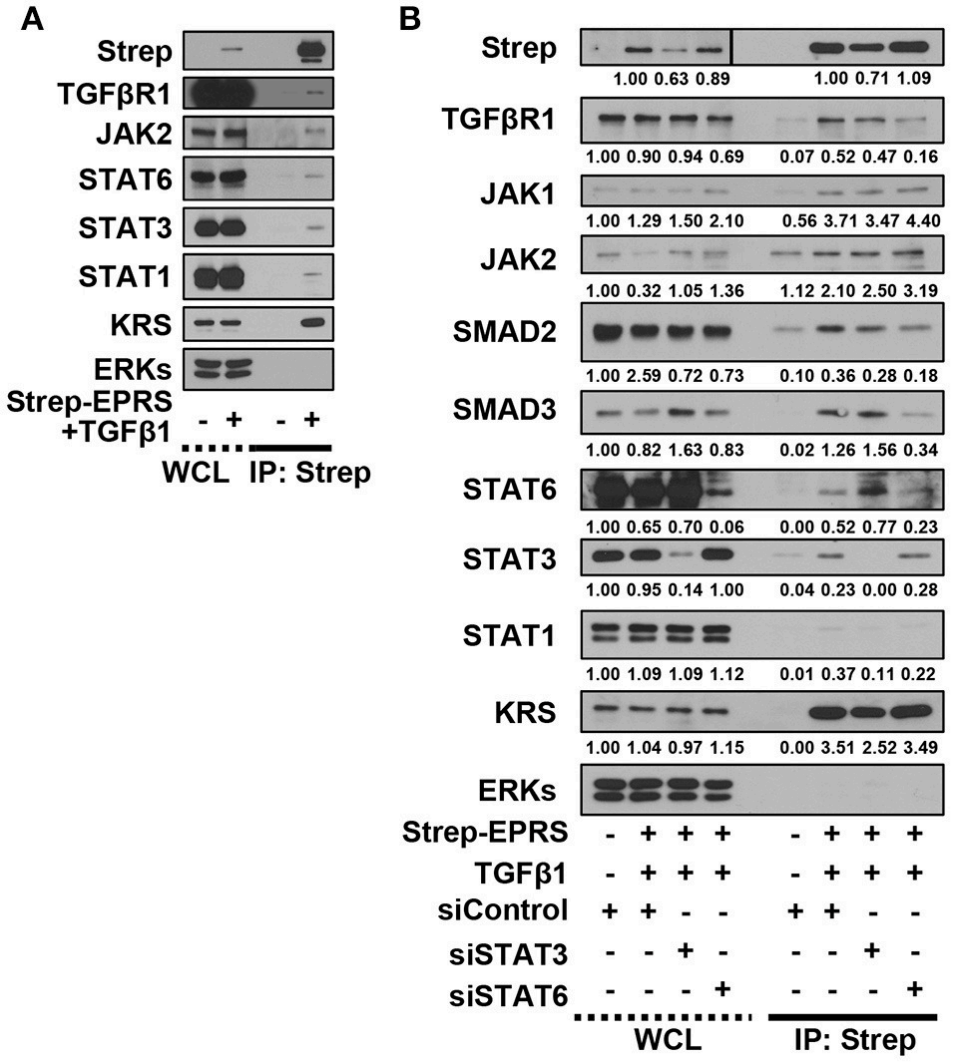
EPRS-mediated signaling in TGFβ1-treated cells involved the formation of a multi-protein complex consisting of STAT6 and TGFβ1R. **(A,B)** A549 cells (–) or transiently-expressing Strep-tagged EPRS (Strep-EPRS, +) cells without **(A)** or with transient transfection of siSTAT3 or siSTAT6 for 48 h **(B)** were treated with vehicle (-) or TGFβ1 (2 ng/ml, +). Whole cell extracts were then prepared and processed for precipitation using streptavidin-agarose beads, and the precipitates were immunoblotted for the indicated molecules. ERKs were immunoblotted for the internal loading controls of the lysates. Data represent three independent experiments.

We then tested for potential protein-protein interactions between TGFβ1 signaling and EPRS that regulate STAT6 phosphorylation. A549 cells containing Streptavidin-tagged EPRS (Strep-EPRS) were treated with or without TGFβ1, prior to precipitation of whole-cell extracts using streptavidin agarose beads for immunoblotting assays. Lysyl-tRNA synthetase (KRS), which forms a multi-aminoacyl-tRNA synthetase complex (MSC) with EPRS (Park et al., 2008), was used as a positive control. In TGFβ1-treated cells, Strep-EPRS transiently precipitated with TGFβR1, JAKs, and STATs, which included STAT6 (Figure 4A). We tested the effect of STAT3 or STAT6 suppression on multi-protein interactions. STAT6 expression was required for the EPRS-mediated multi-protein complex formation (Figure 4B). Specifically, EPRS interaction with TGFβ1R and SMAD2/3 required STAT6 but not STAT3. Interestingly, STAT3 suppression resulted in increased binding of STAT6 to the EPRS/TGFβ1R-containing protein complex (Figure 4B). Interactions between EPRS and JAKs were independent of STAT3 and STAT6 (Figure 4B). Our results suggest that STAT6 is critical for the formation of the multi-protein complex for TGFβ1-induced signaling of ECM protein expression. STAT3 and STAT6 may be involved in parallel signaling pathways to regulate this process.

### Lung tissues from bleomycin-treated mice showed EPRS-dependent STAT6 phosphorylation and ECM protein production *in vivo*

**Figure 5:**
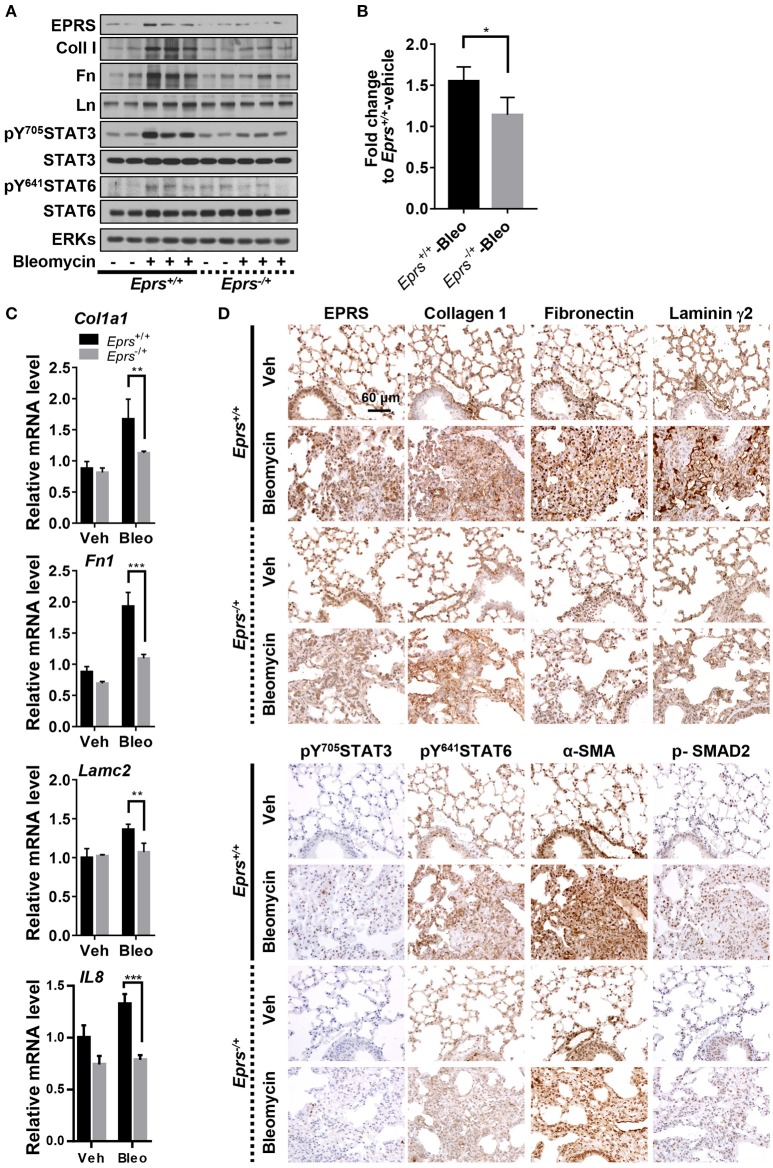
Lung tissues from bleomycin-treated mice showed EPRS-dependent STAT6 phosphorylation and ECM protein production *in vivo*. **(A–D)** Wildtype (WT, *Eprs*^+/+^) and *Eprs*^−/+^ hetero-knockout (KO) C57BL/6 mice were intratracheally treated once with vehicle (*n* = 4 for WT and *n* = 5 for *Eprs*^−/+^ hetero-KO) or bleomycin (1 mg/kg in PBS, *n* = 9 for WT and *n* = 7 for *Eprs*^−/+^ hetero-KO). After 28 days, mice were sacrificed, and lung tissues were collected for analyses. Lung tissue extracts were prepared and processed for immunoblots for the indicated molecules **(A)**, hydroxyproline assays **(B)**, and qRT-PCR for the indicated mRNAs **(C)**. Data are presented at mean ± SD. ^*^, ^**^, and ^***^ indicate significance at *p* < 0.05, 0.01, and 0.001, respectively (calculated by Student's *t*-tests). **(D)** The lung tissues were processed for immunohistochemistry, before image capturing at 40 x. Data represent three different experiments.

To investigate the physiological roles of EPRS in pulmonary fibrosis *in vivo*, WT (*Eprs*^+/+^) and *Eprs*^−/+^ hetero-knockout (KO) mice were treated with bleomycin to induce lung fibrosis by intratracheal instillation before analysis, since homozygous *Eprs*^−/−^ is embryonic lethal. Bleomycin-treated WT *Eprs*^+/+^ mice showed the highest increase in expression of ECM proteins, such as fibronectin, collagen I, and laminins, compared with bleomycin-treated *Eprs*^−/+^ hetero-KO mice and untreated WT mice (Figure 5A). Levels of pY^705^STAT3 and pY^641^STAT6 were also elevated in bleomycin-treated *Eprs*^+/+^ mice, compared with *Eprs*^−/+^ hetero-KO mice (Figure 5A). Our results suggest that STAT6 activation is part of EPRS-dependent signaling for ECM protein expression *in vivo*. We observed a slight upregulation in EPRS expression in bleomycin-treated WT *Eprs*^+/+^ mice, compared with other groups, indicating that EPRS might function as a pro-fibrotic molecule.

Hydroxyproline assays to measure collagen I levels in lung extracts showed that bleomycin-treated *Eprs*^+/+^ mice had higher levels compared with bleomycin-treated *Eprs*^−/+^ mice (Figure 5B). In addition to *IL8*, which is used to characterize IPF (Carre et al., 1991), *Col1a1, Fn1*, and *Lamc2* mRNA levels were also upregulated by bleomycin treatment in *Eprs*^+/+^ lungs but not *Eprs*^−/+^ hetero-KO lungs (Figure 5C).

Lung immunohistochemistry revealed more patchy fibrosis and fibroblastic foci in bleomycin-treated *Eprs*^+/+^ mice compared with *Eprs*^−/+^ mice (Figure 5D). Bleomycin treatment also led to marked increases in collagen I, fibronectin, and laminin γ2 synthesis in *Eprs*^+/+^ mice compared with *Eprs*^−/+^ mice. Levels of phospho-SMAD2, pY^705^STAT3, and pY^641^STAT6, which showed intense nuclear staining, were diminished in *Eprs*^−/+^ mice (Figure 5D). The myofibroblasts marker α-SMA was positive in fibroblastic foci of bleomycin-treated mice lungs. These results suggest that the bleomycin-mediated fibrotic phenotypes in animal lungs are dependent on EPRS expression.

## Discussion

**Figure 6:**
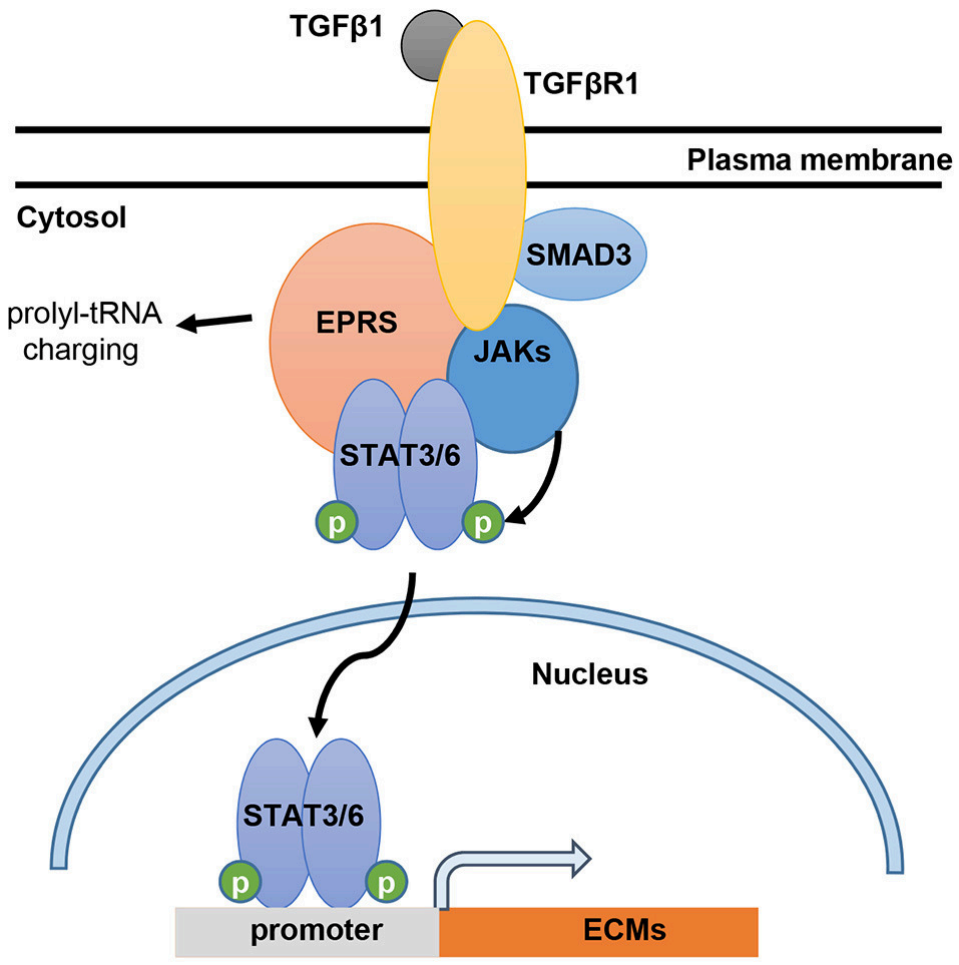
Working model for EPRS-dependent signaling during TGFβ1-mediated expression of ECMs. TGFβ1 binding to TGFβR1 can stimulate formation of a signaling complex consisting of TGFβR1, SMAD3, JAKs, STAT3/6 and EPRS for active JAKs-mediated STAT3/6 phosphorylation. Phosphorylated STAT3/6 can enter the nucleus for transcriptional induction of ECM genes, such as collagen α1 (*COLA1*), laminin γ2 (*LAMC2*), and fibronectin 1 (*FN1*).

In this study, we showed that EPRS regulates the TGFβ1-mediated expression of ECM proteins such as collagen I and fibronectin. Our *in vitro* and *in vivo* analyses demonstrated that the signal for ECM protein synthesis might be transduced via a multi-protein signaling complex composed of TGFβR1, SMAD3, JAKs, and STAT6. EPRS may have functions independent of translational tRNA charging that serve as a signaling molecule for TGFβ1-induced ECM protein synthesis and mesenchymal marker expression, presumably leading to fibrotic phenotypes (Figure 6). Therefore, EPRS is a promising target for anti-IPF therapy.

The binding target of the anti-fibrotic agent, HF, first revealed the link between EPRS and fibrosis (Keller et al., 2012). HF competitively inhibits PRS, which activates the AAR pathway because of naked-tRNA accumulation. HF-mediated inhibition of PRS also cause decreased ECM expression, which is overcame by exogenous proline treatment, indicating that PRS can be involved in ECM translation via tRNA charging with proline (Keller et al., 2012). However, it cannot be ruled out that PRS play roles in ECM expression at non-translational processes, since variable ECMs can be composed with different levels of proline. Additionally, this previous study had not shown EPRS regulation of TGFβ1-induced ECM protein synthesis. Moreover, although a previous study showed a fibrotic role of EPRS in a lung fibroblast IMR90 cell line, here we found that alveolar type II epithelial cells may lead to the formation of fibrotic foci in IPF under the influence of TGFβ1. TGFβ1 is a master regulator of fibrosis that induces epithelial to mesenchymal transition (EMT) and analyzing its role in EPRS-mediated ECM regulation is critical for developing IPF treatments.

Idiopathic pulmonary fibrosis (IPF) (Lederer and Martinez, 2018) is currently managed with nintedanib and pirfenidone. These drugs slow down the rate of forced vital capacity decline by ~50% over 1-year period (Lederer and Martinez, 2018) but do not completely cure the disease. The anti-fibrotic reagent, HF, causes significant side effects including severe gastrointestinal lesions and hemorrhage (Jiang et al., 2005). Novel and safe treatment methods for IPF are therefore needed. Recent studies have begun uncovering the mechanisms of IPF pathogenesis. STAT6-mediated signaling is important for the development of carbon nanotube-induced fibrotic lung disease (Nikota et al., 2017). The JAK2/STAT3 pathway is activated in IPF, and treatment with JSI-124 (a dual inhibitor of JAK2/STAT3) decreases collagen deposition during lung fibrosis (Milara et al., 2018). In the present study, we used *in vitro* and *in vivo* models to reveal a novel relationship between EPRS and STAT6 and their participation in IPF.

In studying TGFβ1-induced activation of the STAT signaling cascade for ECM protein synthesis, we found that EPRS was an upstream regulator of STAT3/6 activation. TGFβ1-induced SMAD3 activation was important for activating STAT6, which was critical for formation of the multi-protein complex of TGFβR1, EPRS, JAKs, and STAT3/6. Both STAT3 and STAT6 appear to act downstream of TGFβ1 stimulation, possibly in parallel signaling pathways. However, STAT3 suppression led to higher levels of STAT6 in the multi-protein complex, indicating that STAT6 was more important than STAT3 for TGFβ1-induced, EPRS-mediated ECM protein synthesis. Previous studies have shown that EPRS forms complexes with other proteins. EPRS translocates to the cell surface to bind to TGFβ1R. Phosphorylation of EPRS at Ser999 causes it to dissociate from the MSC and translocate to the membrane where it interacts with the fatty-acid transporter, FATP1, upon insulin stimulation of adipocytes (Arif et al., 2017). TGFβ1 treatment induces TGFβR1-JAK1 and STAT3-SMAD3 to form a protein complex (Tang et al., 2017). These studies validate our results regarding the EPRS-containing multi-protein complex. We also found that suppression of STAT6 but not of STAT3 abolished the formation of a complex between EPRS, TGFβR1, and SMAD2/3. Our findings suggest that EPRS is a novel component of the TGFβR1-JAK complex and STAT6 is critical for the formation of the EPRS-TGFβR1-JAKs-STATs multi-protein signaling complex that mediates ECM synthesis.

Our *in vivo* animal studies showed that ERPS protein levels were slightly upregulated in bleomycin-treated WT *Eprs*^+/+^ mice compared with *Eprs*^−/+^ KO mice. However, EPRS mRNA levels were upregulated 2.5-fold in TGFβ1-treated A549 cells compared with control cells, although EPRS protein levels were unchanged (Figure 1C). These differences in EPRS mRNA and protein levels might be the result of variable treatment times between the *in vitro* and *in vivo* experiments (1 vs. 28 days). Because IPF is a chronic disease, EPRS might be upregulated during the development of fibrosis, as seen in our *in vivo* experimental model.

In conclusion, our study showed that EPRS might be a signaling molecule underlying TGFβ1-induced ECM protein synthesis and is a promising potential target for the treatment of IPF.

## Author contributions

D-GS performed most experiments and wrote the 1st version of manuscript. DK: helped with animal study, JHK and SK helped with experimental reagents. JWJ, SHN, JEK, and H-JK helped with imaging experiments or with reagents. C-HP, SK, and JWL discussed the data and JWL wrote the manuscript.

### Conflict of interest statement

The authors declare that the research was conducted in the absence of any commercial or financial relationships that could be construed as a potential conflict of interest. The handling editor declared a shared department with several of the authors (D-GS, DK, JWJ, SHN, JEK, H-JK, JHK, SK and JWL) at time of review.

## Abbreviations

α-SMA: α-smooth muscle actin
AAR: amino acid starvation response
ECM: extracellular matrix
EPRS: glutamyl-prolyl-tRNA-synthetase
ERS: glutamyl-tRNA-synthetase
JAKs: Janus kinases
PRS: prolyl-tRNA-synthetase
STAT: Signal transducer and activator of transcription.
