# Supplementary material for: Glutamyl-Prolyl-tRNA Synthetase Regulates Epithelial Expression of Mesenchymal Markers and Extracellular Matrix Proteins: Implications for Idiopathic Pulmonary Fibrosis
Source: Front Pharmacol. 2018 Nov 20;9:1337. doi: 10.3389/fphar.2018.01337 (PMC6256097; doi:10.3389/fphar.2018.01337)

Glutamyl-Prolyl-tRNA Synthetase Regulates Epithelial Expression of Mesenchymal Markers and Extracellular Matrix Proteins: Implications for Idiopathic Pulmonary Fibrosis

Dae-Geun Song[1,2], Doyeun Kim[3], Jae Woo Jung[4], Seo Hee Nam[1], Ji Eon Kim[1], Hye-Jin Kim[1], Jong Hyun Kim[3], Cheol-Ho Pan[2], Sunghoon Kim[3], and Jung Weon Lee[1,3,*]

Figure S1. Supplementary unprocessed immunoblots used in this study are included.

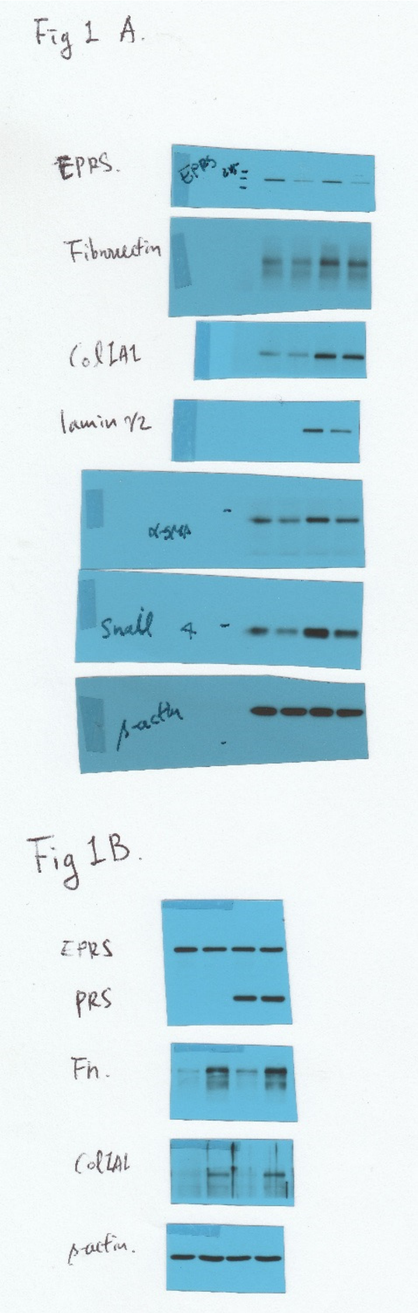

Fig. 1C

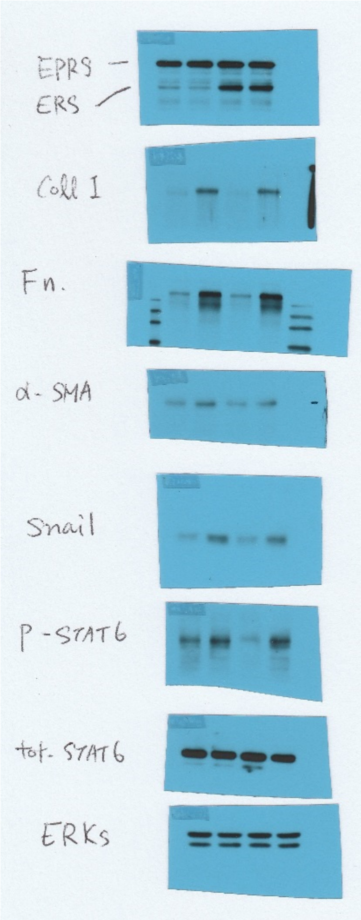

Figure S1-continued.

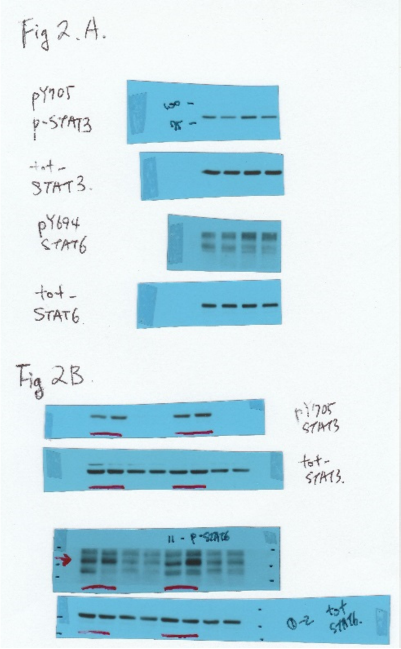

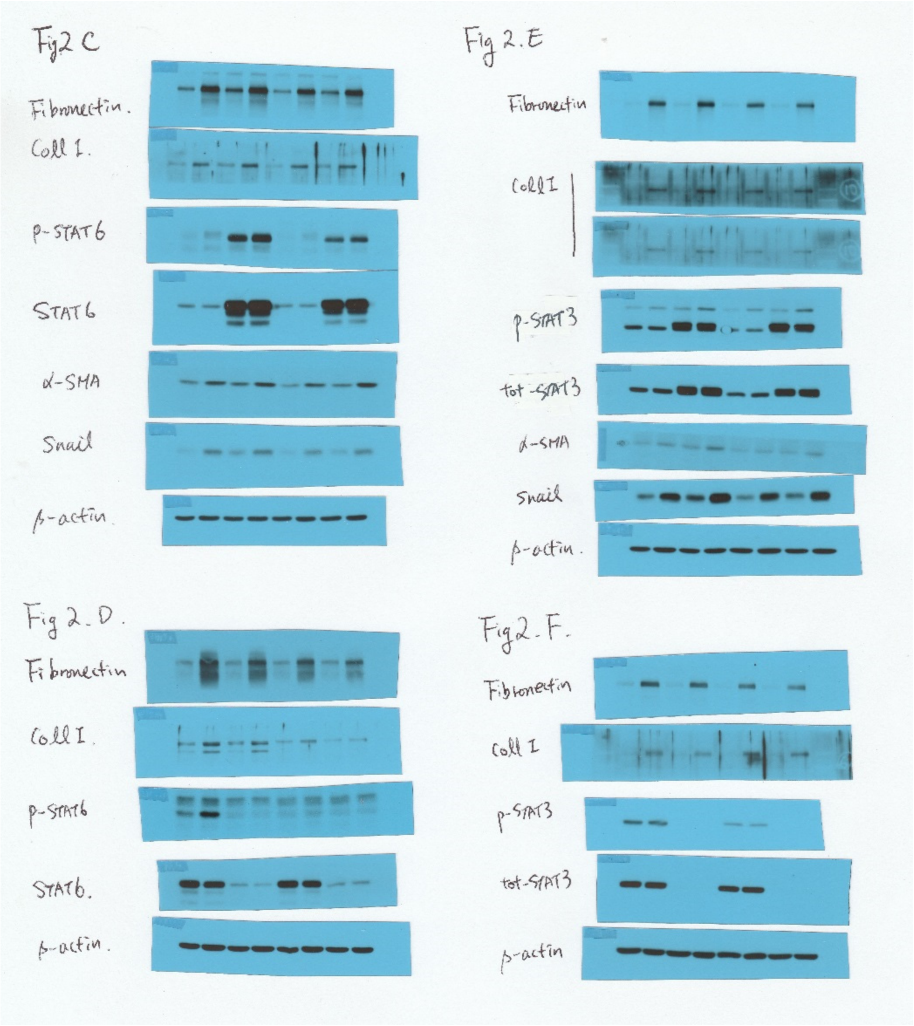

Figure S1-continued.

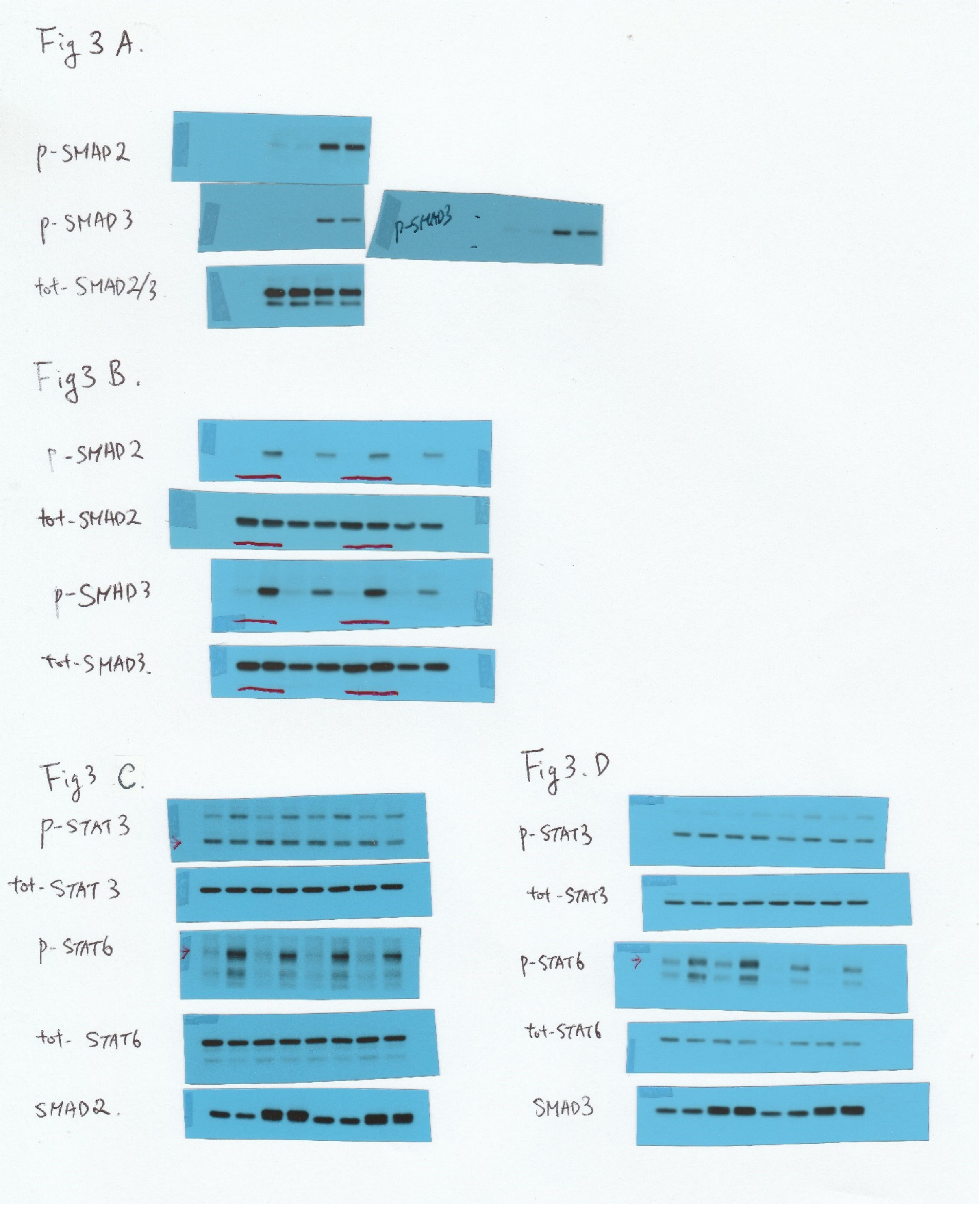

Figure S1-continued.

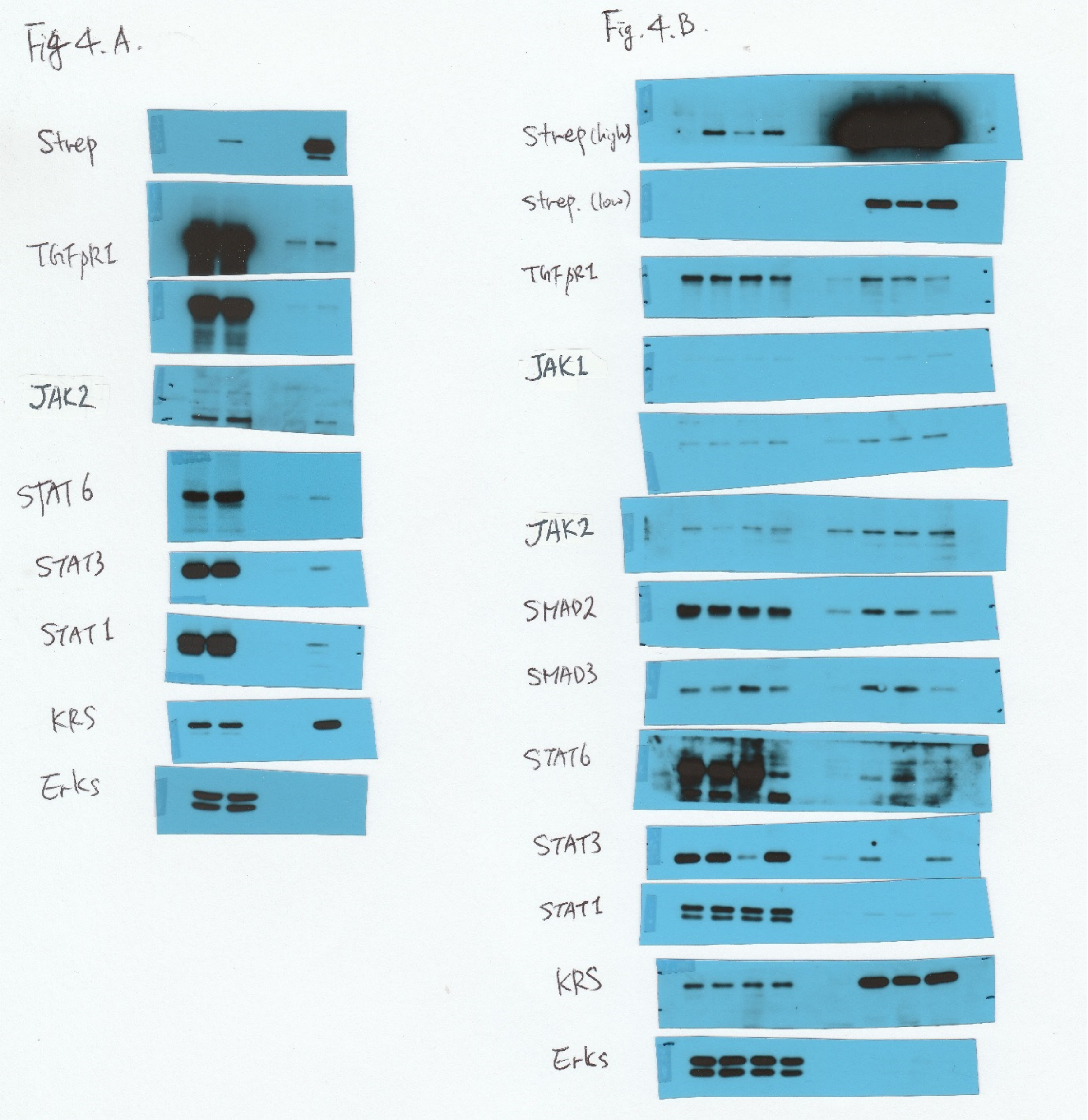

Figure S1-continued.

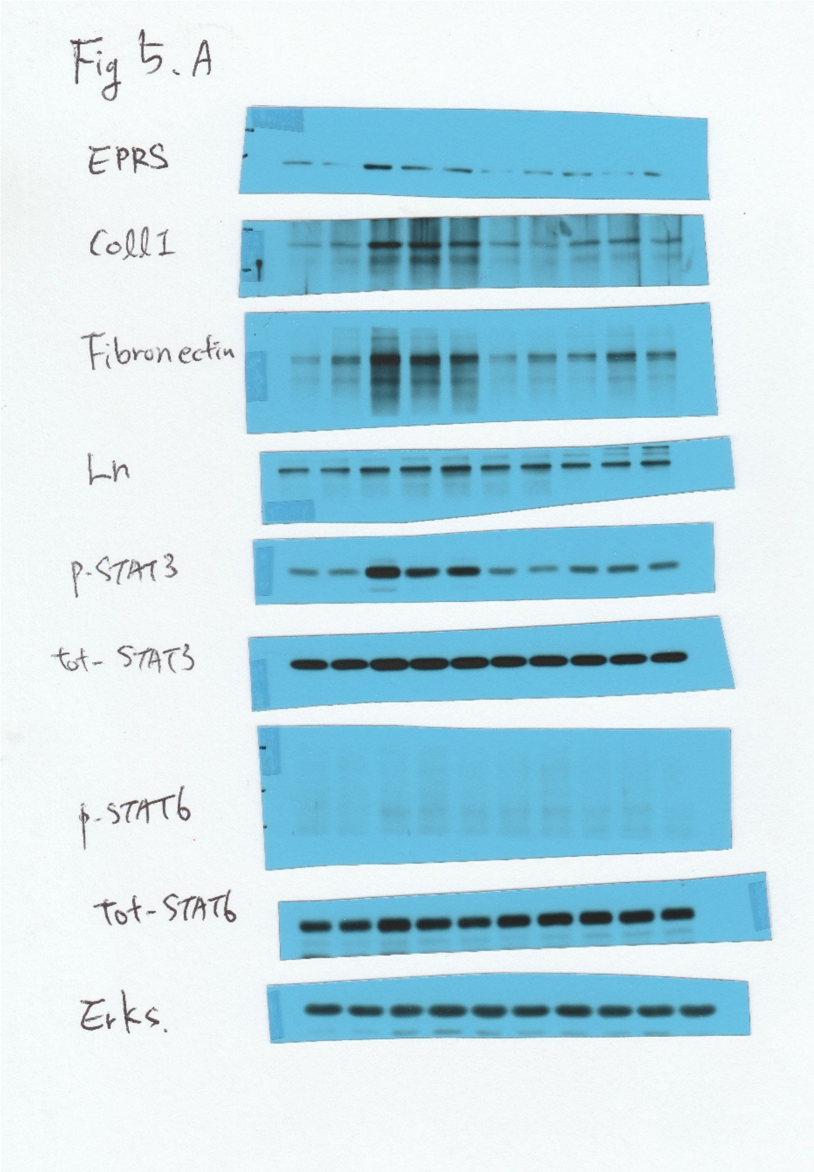

Supplement: Supplementary file 1 [file Data_Sheet_1.pdf]
